# Resilience and Preparedness Across Place: A Multilevel Analysis of Urban–Rural and Socioeconomic Divides

**DOI:** 10.1111/risa.70155

**Published:** 2025-11-21

**Authors:** Ebba Henrekson, Susanne Wallman Lundåsen

**Affiliations:** ^1^ Center for Civil Society Research Marie Cederschiöld University Stockholm Sweden; ^2^ Center for Educational Leadership and Excellence Stockholm School of Economics Stockholm Sweden; ^3^ Centre for Local Government Studies Linköping University Norrköping Sweden

## Abstract

This study investigates how local context—specifically urban versus rural environments and socioeconomic conditions—influences individual crisis preparedness and resilience in Sweden. Using multilevel survey data from 12,574 respondents, we analyze both proactive preparedness actions and perceived resilience. Results show that rural residents report higher levels of preparedness and resilience than their urban counterparts. However, these differences in preparedness attenuate when controlling for individual risk perception, suggesting a mediating role. Socioeconomic context, on the other hand, does not show an independent effect beyond individual characteristics, indicating compositional rather than contextual influences. The findings highlight the importance of tailoring crisis preparedness strategies to both individual and local characteristics and stress the need for authorities to consider spatial disparities in vulnerability when planning for future crises.

## 1 Introduction

In a world increasingly marked by extreme weather events and widespread technological disasters, understanding vulnerability and resilience among residents across communities has become particularly important. It is crucial to explore both the potential extent of damage that various hazards might cause and the capacity of communities to respond and recover (Cutter et al. 2003; Ran et al. 2020; Spielman et al. 2020).

Two key concepts in this context are preparedness and resilience, which together help assess not only the risks that groups and communities face but also how well they are equipped to cope with and recover from such challenges. By *preparedness*, we refer to the proactive actions individuals take to equip themselves for potential crises—such as stockpiling food and water or making emergency plans. *Resilience*, in turn, is understood as the capacity to endure a crisis without immediate access to essential public services like electricity, running water, or groceries.

In contemporary discourse, there has been a marked shift toward emphasizing individual responsibility in crisis preparedness (Rådestad and Larsson 2020; Nilson and Wester 2025). This shift is grounded in integrated approaches to risk, crisis, and emergency management that stress the interplay between individual security, risk assessment, and vital societal functions (e.g., Bergstrand et al. 2015; ‘t Hart and Sundelius 2013; Norris et al. 2008). As governments increasingly acknowledge the limits of institutional crisis response capacities, national strategies have placed growing emphasis on public awareness and self‐preparation (Cutter et al. 2010; ‘t Hart and Sundelius 2013; Kvarnlöf 2020), effectively promoting the responsibilization of individuals (Rådestad and Larsson 2020).

The practical consequences of this responsibilization are most visible when institutional support is delayed, as citizens are expected to manage initial disruptions independently (Norris et al. 2008; Orru et al. 2025; Ran et al. 2020; Spielman et al. 2020). Policy shifts now reflect the expectation that individuals take proactive roles in preparing for crises—by stockpiling essential supplies, staying informed, and creating emergency plans. Sweden offers a clear example of this trend: while earlier guidance from the Swedish Civil Contingencies Agency recommended that residents be self‐sufficient for 3 days in the event of a major crisis or disaster, this threshold was extended to at least 1 week in 2018, underscoring the growing demand for individual preparedness to support broader societal resilience (Kvarnlöf 2020, 2021).

Despite growing recognition of how individual preparedness varies across social groups (e.g., Bolin 2007; Cutter et al. 2003; Orru et al. 2025; Ran et al. 2020), the importance of geographical differences and residential segregation for resilience and preparedness remains largely underexplored within the Swedish context. Still, geographical locations matter. This is emphasized by the fact that resources, engagement, and social networks depend on local contexts, and research clearly shows that these factors are unevenly distributed across different geographical areas even in relatively equal countries like Sweden (e.g., Dahlberg et al. 2024; Mutgan and Mijs 2023). These spatial inequalities have become increasingly significant as Sweden has undergone notable social and demographic changes in recent decades. The population has grown more diverse, with the share of first‐generation immigrants rising from about 10% in 2000 to 20% by 2024. Following from this, an overarching trend that accentuates differences between places is the increasing socioeconomic and ethnic residential segregation occurring primarily in cities (Mutgan and Mijs 2023). This could complicate the responsibilization of individuals during disasters and crises through, for instance, language and cultural barriers (Carlberg et al. 2022). Moreover, there are considerable differences in physical distances to important services across urban and remote rural areas that may impact the willingness to prepare and levels of resilience across contexts.

The COVID‐19 pandemic was an example of how local contexts mattered not only in terms of the capacity of local government institutions but also in terms of the capacity of residents to be prepared to act collectively in a moment of crisis (Wollebæk et al. 2022). The pandemic highlighted marked local variation in first‐phase mortality and later responses to guidance—such as vaccine uptake—in Sweden as well as in many other contexts (Brandén et al. 2020; Carlberg et al. 2022; Mankell and Abdelzadeh 2023).

There are also important differences across sociodemographic groups regarding risk‐perceptions and the ability to deal with risks. Nilson and Wester (2025) observe that individuals with lower socioeconomic status tend to perceive heightened risk across a broad range of societal domains. This heightened sense of vulnerability can make it difficult for groups to prioritize between many different risks, and paradoxically, those who perceive the most risks may be the least prepared, overwhelmed by the sheer number of potential threats. These differences in risk perception have important consequences. First, they affect households’ capacity to prepare for and cope with crises (Bergstrand et al. 2015; Norris et al. 2008; Ran et al. 2020). Second, when disadvantaged groups with lower preparedness are geographically concentrated, they contribute to spatially embedded vulnerabilities (Cong et al. 2023; Cutter et al. 2008; Laska and Morrow 2006; Norris et al. 2008; Ran et al. 2020; Spielman et al. 2020). In this way, differences in risk awareness can deepen preexisting social inequalities during a crisis.

Against this backdrop, it is important to investigate differences in preparedness and resilience across socioeconomic groups and local contexts. The aim of this study is to examine how individual preparedness and resilience in the face of crises are shaped by personal characteristics and by local contexts defined by both urban–rural location and neighborhood socioeconomic conditions. Our research is guided by the following questions:

- How do levels of preparedness and resilience vary between urban and rural areas?
- How do they vary between neighborhoods with different socioeconomic profiles?

Understanding potential heterogeneities in preparedness and resilience levels within local populations is crucial for both local and national decision‐makers. Insights from this study can inform the allocation of resources, enhance crisis communication strategies, and support the development of tailored interventions in high‐risk or underserved communities.

To address the aim of this article, we analyze representative survey data collected from a random sample of Swedish residents across different local contexts. The broad and imprecise nature of terms like “rural” and “urban” is illustrated by the fact that, depending on the definition used, anywhere from 13% to 76% of Sweden's population can be classified as living in a rural area (Tillväxtverket 2024). Although many definitions of “rural” and “urban” exist, statistical analysis requires a clear classification; here, we follow Statistics Sweden's definition that classifies areas at the DeSO level—that is, very local, neighborhood‐scale statistical units. In addition, we use the Swedish Segregation Barometer to categorize neighborhoods into five groups based on levels of socioeconomic advantage and disadvantage.

Sweden represents a compelling case for this inquiry. On the one hand, its long‐standing commitment to egalitarian policies, aimed at minimizing disparities between regions and individuals, suggests we might expect only small differences in preparedness between residents of different types of local communities. On the other hand, mounting empirical evidence reveals non‐negligible differences in living conditions and attitudes across district types within Sweden, driven by demographic and socioeconomic disparities (e.g., Dahlberg et al. 2024; Erlingsson et al. 2023; Guldåker et al. 2021; Henrekson et al. 2025; Mutgan and Mijs 2023; Nilson and Wester 2025).

## 2 Individual Factors Influencing Preparedness and Resilience

Research on disaster preparedness and resilience highlights the importance of both individual and contextual factors. We begin by reviewing studies that emphasize individual‐level characteristics, before turning to the literature on how broader social and geographic contexts shape resilience. Individual resources and past experiences of crises and disasters may significantly influence levels of preparedness (Bolin 2007; Enander et al. 2015; Ran et al. 2020). For example, groups that have experienced previous disasters like severe storms, hurricanes, or wildfires are often more prone to prepare compared to groups without such experiences (Bolin 2007; Enander et al. 2015).

These experiences may also shape how risks are perceived, which in turn has been shown to affect preparedness decisions (Olofsson and Rashid 2011). Groups with a heightened sense of risk are generally more prone to take steps to become prepared (Enander et al. 2015). Moreover, studies have found that perceptions of societal risks vary significantly across social groups; for instance, white men often underestimate risks compared to other groups, a phenomenon extensively documented (Finucane et al. 2000; Olofsson and Rashid 2011; Palmer 2003). Furthermore, Nilson and Wester (2025) observed that individuals with lower socioeconomic status are likely to perceive a heightened sense of risk across many societal domains. They conclude that groups perceiving risks in multiple areas may struggle to prioritize among them, which in turn can overwhelm individuals and paradoxically result in lower levels of crisis preparedness.

Similar to risk perceptions, studies have found that individual crisis preparedness also varies according to socioeconomic status and that these differences occur even within a relatively equal country like Sweden (Eriksson and Denk 2024; Guldåker 2009; Kim and Kim 2022; Kvarnlöf 2021). Groups with higher levels of education tend to be more involved in crisis preparedness, as are individuals living with a partner compared to those living alone. Type of housing is also associated with preparedness: Homeowners are generally more prepared for crisis than those living in rental housing (Eriksson and Denk 2024; McCarthy and Friedman 2023). Taken together, these results indicate that socioeconomic resources are significantly associated with crisis preparedness, both in Sweden and in other comparable contexts.

Furthermore, studies show that perceptions of resilience—the extent to which individuals believe they could endure a disaster—also differ across demographic groups. Men and younger individuals generally rate themselves as more capable of coping than women and older individuals (Norris et al. 2008; Saja et al. 2021). Concurrently, evidence from low‐income settings calls into question the hypothesis that disadvantaged groups are invariably less resilient following disasters. In Bangladesh, a study of households affected by a cyclone showed that poorer households, despite greater vulnerability, at times demonstrated stronger coping and recovery capacities than wealthier households (Akter and Mallick 2013).

Different types of social inequalities often intersect; for example, ethnic minority groups more frequently have lower socioeconomic status and more frequently live in low‐income neighborhoods (Bolin 2007). Previous studies highlight the importance of preparedness planning that considers existing social inequalities (Bolin 2007; de Oliveira Mendes 2009; Ran et al. 2020). Even where immigrant groups possess considerable crisis experience such as being a refugee from a country involved in an armed conflict, the penetration of Swedish authorities’ crisis messages is often low, producing preparedness gaps in these groups attributable to communication shortfalls (Olofsson 2007). These communication shortfalls have material consequences during response operations: they can heighten risk for affected populations and, because vulnerability is socially and spatially interconnected, increase the community's overall vulnerability (Bergstrand et al. 2015; Bolin 2007; de Oliveira Mendes 2009). Moreover, previous research shows that conflict‐driven forced migration is often associated with elevated risks of mental health problems such as depression and post‐traumatic stress disorder, which may influence risk perception and preparedness behaviors (Siriwardhana et al. 2014; Turrini et al. 2017).

## 3 The Importance of Local Community Factors for Preparedness and Resilience

### 3.1 Residential Segregation and Inequality

Resources are distributed inequitably not only across social groups but also geographically, both nationally and within regions or cities (Dahlberg et al. 2024). Residential segregation contributes significantly to neighborhood‐level disparities in average income and education. Such segregation is often identified as a persistent factor that entrenches inequalities by restricting access to resources, opportunities, and social networks (Sampson and Levy 2020).

Given the impact of residential segregation on other types of social outcomes (Mutgan and Mijs 2023; Sampson and Levy 2020), it is plausible to assume that segregation also affects preparedness levels. Preparedness and resilience depend on both individual factors and the social and institutional environments—households, neighborhoods, and municipalities—in which people live; accordingly, disasters frequently strike economically disadvantaged regions more severely than affluent ones (Oliver‐Smith 1986, 1996). Economically disadvantaged areas bear a disproportionate burden driven not only by heightened exposure to hazards—for instance, through substandard housing and infrastructure—but also by institutional limitations, including weaker governance structures (Ran et al. 2020).

Norris et al. (2008) draw on the concepts of robustness, redundancy, and rapid response to outline four key types of interconnected adaptive capacities that support a community's recovery in the face of major stressors: economic development, social capital, information and communication, and community competence. When it comes to economic development, crucial factors include not only the overall availability and variety of economic resources but also how equitably these resources are distributed within the community. Communities differ in vulnerability depending on their resource levels, a condition captured by the concept of “place vulnerability,” which integrates geographical and physical contexts with social vulnerabilities (Oliver‐Smith 1986; de Oliveira Mendes 2009). Communities rich in resources often have greater redundancy of resources, enabling their members to better cope during disasters or emergencies (Bergstrand et al. 2015). Conversely, empirical evidence shows that structurally disadvantaged communities typically lack such redundancy, facing greater barriers to postcrisis recovery—constraints rooted in systemic conditions rather than community deficiencies (Bergstrand et al. 2015; Ran et al. 2020).

The interplay between places and individuals is examined in a Canadian study by Yong et al. (2020). The authors identified significant variations in disaster preparedness across social groups; the relationship between local context and preparedness differed notably. For instance, immigrant groups exhibited lower preparedness when living in communities characterized by high social trust—a pattern not observed among Canadian‐born respondents. These results suggest that different groups may interpret and respond to identical social cues in varying ways. Thus, it is crucial to account for specific social and cultural contexts when identifying potential place vulnerabilities.

### 3.2 Urban and Rural Contexts

Another aspect related to the local contexts that has been shown to be important for levels of preparedness and resilience is the degree of urbanity or rurality (Eriksson and Denk 2024; Guldåker 2009). Whether urban or rural contexts are considered more or less vulnerable to disasters varies across country contexts (Norris et al. 2008). Even in a highly developed context like Sweden, rural communities tend to be more attuned to self‐reliance during emergencies and to have experience of disruptions such as power outages (Guldåker 2009). Consequently, residents in rural areas are more likely to have alternative sources of power, heating, and water in case of a crisis.

Urban and rural areas often exhibit additional differences. For instance, residents of rural communities in Sweden, especially those in sparsely populated areas, face significantly longer physical distances to critical institutions, such as hospitals and police stations (Larsson 2021; Stenbacka 2022). These geographic disparities require adaptations and cooperation among local residents to manage the associated risks (Stenbacka 2022). Consequently, rural residents may be less likely to expect immediate emergency support during a crisis and are thus more inclined to prepare independently (Guldåker 2009).

Urban and rural contexts also differ structurally. In Sweden—as in many Western countries—rural areas typically have older age profiles and lower ethnic heterogeneity, creating distinct risk profiles; for example, longer distances to healthcare disproportionately burden older adult residents. In contrast, vulnerability in urban districts is closely associated with socioeconomic disadvantage, residential segregation, and ethnic minority status (Gustafsson et al. 2017). Even within Scandinavian welfare states, community trust varies with neighborhood socioeconomic status and segregation (Dinesen et al. 2020; Ivarsflaten and Strømsnes 2013; Wallman Lundåsen and Wollebæk 2013; Wallman Lundåsen 2023). Urban neighborhoods marked by concentrated low‐income neighborhoods and ethnic segregation often show lower trust than rural or more affluent urban areas, which can hinder local cooperation during crises (Dinesen et al. 2020; Sampson 2017). Conversely, more advantaged urban neighborhoods tend to exhibit higher collective efficacy and benefit from greater private and redundant resources, enhancing resilience (Sampson and Levy 2020).

Another important structural difference between rural and urban communities lies in the types of housing. Rural residents often live in single‐family homes, which typically offer more space that can, for instance, be used for storage or shelter. In contrast, residents in densely populated urban areas are more likely to live in apartments with limited storage capacity, making it, for instance, harder to stockpile supplies. Moreover, disadvantaged urban neighborhoods often experience overcrowding, which is often associated with a range of negative consequences for residents (Lorentzen et al. 2024). Therefore, we argue that it is important to recognize that individuals are embedded in diverse local contexts that vary not only in socioeconomic status but also in their degree of urbanity or rurality.

In sum, this study is grounded in the expectation that both individual and contextual factors shape levels of crisis preparedness and resilience. We approach socioeconomic resources as both an individual and contextual factor. At the individual level, resources, such as income, education, and social capital, can vary substantially (Dinesen et al. 2020; Wallman Lundåsen and Wollebæk 2013), influencing individual households’ ability to prepare for and cope with disruptions. At the contextual level, socioeconomic resources and social capital shape local environments, especially under conditions of residential segregation, where people with similar socioeconomic status tend to cluster in the same areas. This can produce “wealthier” and “poorer” neighborhoods, which differ in terms of resource redundancy and collective capacity to recover from crises (Bolin 2007; Cutter et al. 2003). These differences can intersect with the urban–rural divide, as levels of preparedness and resilience may also vary depending on the degree of urbanity or rurality. As both the individual and context levels are theoretically important, we examine whether local contexts have an independent association with preparedness while simultaneously accounting for individual‐level resources. In order to investigate the importance of individual and contextual factors, we apply multilevel models, which allow us to assess variation across both individual and contextual levels.

## 4 Data and Methods

To achieve the aim of this article, we draw on data from the 2024 Trust Barometer survey. The respondents were selected from both a panel and a nationally representative sample. The panel, initiated in 2017, conducted its second wave in 2020 and its third wave in 2024. In 2024, 9993 individuals from the panel participated, yielding a response rate of 71.7%, whereas 2971 individuals from the nationally representative sample responded, with a response rate of 29.7%. The survey, distributed by Statistics Sweden, was available in both paper and web‐based formats, with three reminders sent to nonrespondents. All participants were informed about the purpose of the study and gave their consent to participate in accordance with ethical guidelines. The questionnaire covered a wide range of topics, including trust, safety, and welfare, as well as questions on risk perception, perceived resilience, and individual preparedness. The questions pertaining to resilience and preparedness were based on questions used by the United States Federal Emergency Management Agency, adapted to the Swedish context. Additionally, Statistics Sweden added register data on demographic and socioeconomic factors such as gender, income, and marital status for each respondent. For the current study, we use a subset of 12,574 respondents with no missing data for any variables included in the study. Figure A1 in the Supporting Information section illustrates the structure of the data.

We analyzed our data using two distinct multilevel linear regression models, each addressing different dimensions of individual preparedness. The first model examines factors influencing how long respondents estimate they could manage without access to various types of public infrastructure. The second model explores the factors that affect the specific actions respondents have taken to prepare for disasters and crises.

### 4.1 Dependent Variables

In the models, we use two different dependent variables: a Resilience Index and a Preparedness Index.

The Resilience Index captures the respondents’ perceived ability to manage without public services—an operationalization of the concept of resilience as defined earlier. It is based on four survey items that measure how long the respondents estimate that they would manage without access to running water, electricity, the ability to purchase groceries, and a scenario in which all public services were shut down. The respondents answered using six different options ranging from less than 3 days to more than 3 months. The index was constructed by calculating the mean of four variables. Missing values were handled by imputing values by averaging available responses. The index shows good internal consistency (Cronbach's *α* = 0.855), supporting the treatment of the items as a single scale; the scale mean is 2.68 (SD = 1.18).

**FIGURE 1:**
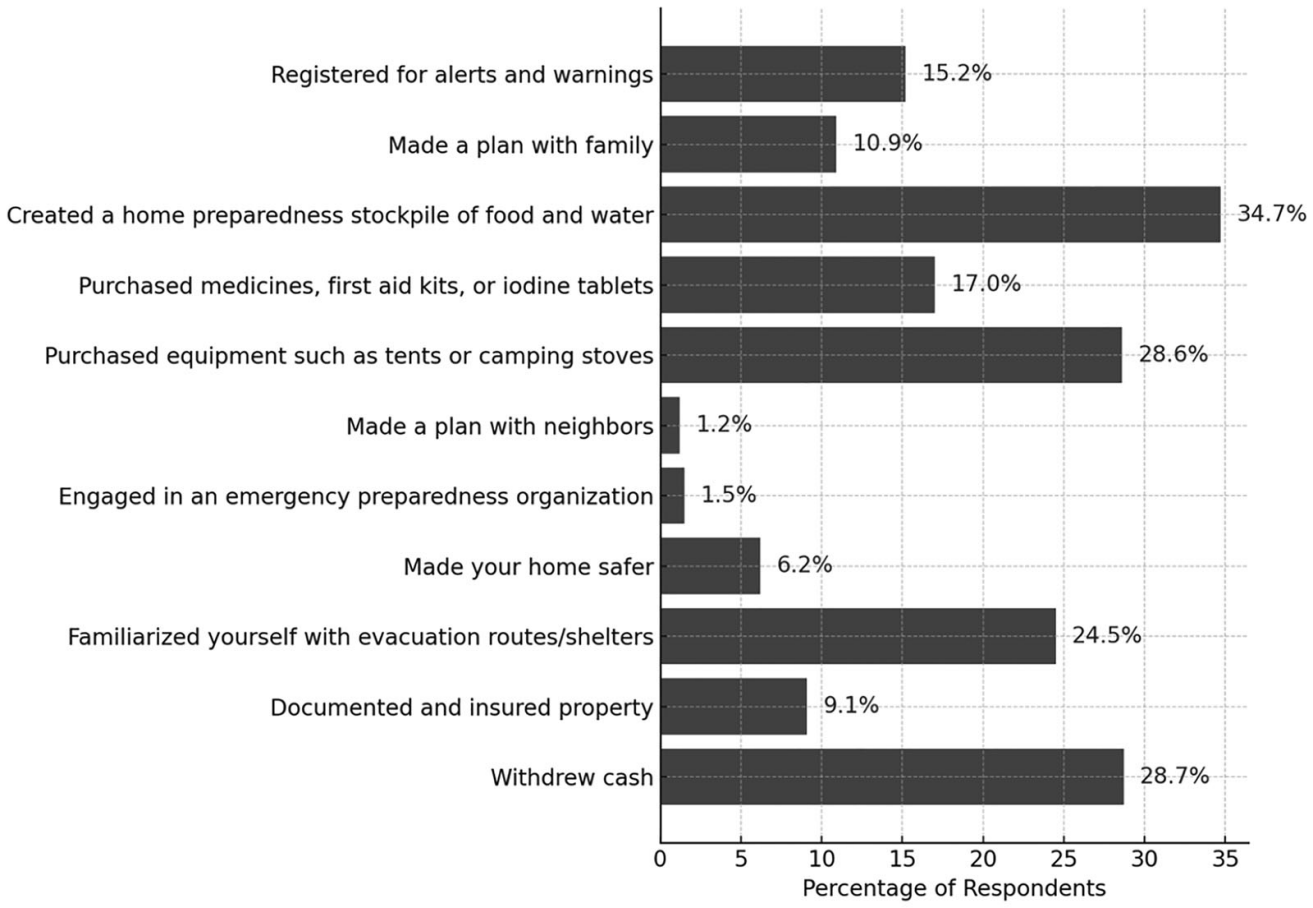
Percentage of respondents that have taken various types of actions to prepare for crisis or disaster.

The Preparedness Index reflects concrete individual actions taken to prepare for crises. It was constructed by summing responses to 11 yes/no questions, with each “yes” (coded as 1) indicating a preparedness action taken by the respondent. As personal preparedness includes both plan‐making and stockpiling (Kohn et al. 2012), the questions encompassed a wide range of actions, such as purchasing equipment (e.g., tents or camping stoves), developing a plan with neighbors, withdrawing cash, and creating a home preparedness stockpile of food and water. Figure 1 presents the percentage of respondents who reported undertaking each of these actions. Notably, 36.6% of respondents had not engaged in any of the actions included in the index. The index has a mean of 1.78 and a standard deviation of 1.92, reflecting a variation in preparedness levels among the respondents.

### 4.2 Independent Contextual Variables

Given the aim of our article, we use two variables related to the context in which respondents reside as our main independent variables. These variables are based on DeSO and RegSO areas. DeSO (abbreviation for Demografiska statistikområden) are small geographic units in Sweden designed by Statistics Sweden for detailed demographic and socioeconomic statistics, smaller than municipalities. Sweden has 5984 DeSO districts, each varying between 700 and 2700 residents. RegSO (abbreviation for Regionala statistikområden, i.e., district statistical units) are larger areas that group multiple DeSO areas and often correspond to a neighborhood or district that citizens recognize.

The first main independent variable measures the rurality/urbanity of the respondents’ place of residence and was constructed based on DeSO areas. It classifies respondents’ places of residence into four categories: (1) rural areas (17.9% of respondents), (2) towns or agglomerations that are not municipal centers (7.3%), (3) municipal centers (45.5%), and (4) the metropolitan areas of Stockholm, Gothenburg, and Malmö (29.3%).

The second main independent variable is based on socioeconomic segregation. The classification is based on the Segregation Barometer, a tool developed by the Swedish National Board of Building, Housing, and Planning. The Segregation Barometer categorizes residential districts into five distinct groups based on three key indicators: the proportion of residents aged 20–64 with a pre‐high school education, the proportion of residents with a low economic standard, and the proportion of residents who have received welfare assistance from the social services for at least 10 months and/or have been unemployed for more than 6 months. This classification divides RegSO areas into five categories based on socioeconomic characteristics: (1) areas with significant socioeconomic challenges (3.2% of respondents), (2) areas with moderate challenges (5.2%), (3) areas with mixed socioeconomic conditions (23.2%), (4) areas with favorable socioeconomic conditions (57.8%), and (5) areas with very favorable socioeconomic conditions (10.6%).

### 4.3 Risk Perception Factors as Individual Level Independent Variables

As we expect risk perceptions to influence levels of preparedness and resilience, we consider a broad array of risks that respondents evaluate for their likelihood of impact. An exploratory factor analysis was conducted to identify underlying dimensions of risk perception related to various types of disasters, including pandemics, wildfires, floods, extreme heat, prolonged power outages, and armed attacks on Sweden. The suitability of the data for factor analysis was confirmed using the Kaiser–Meyer–Olkin (KMO) measure of sampling adequacy and Bartlett's test of sphericity. The overall KMO value was 0.82, with individual values ranging from 0.79 (floods) to 0.87 (pandemics), indicating good sampling adequacy. Bartlett's test (*p* < 0.001) showed significant correlations among the variables, further supporting the use of factor analysis. The two extracted factors explained 48.23% of the total variance, with the first factor accounting for 39.99% and the second factor accounting for 8.24%. Principal axis factoring was employed as the extraction method to identify the underlying factors in the data. Two factors were extracted based on the Kaiser criterion (eigenvalues > 1), and a varimax rotation was applied to achieve a clearer interpretation of the factor structure.

The factor scores were calculated using the regression method, which produces scores based on a weighted combination of the variables associated with each factor. This method ensures that the factor scores are uncorrelated and optimally represent the latent constructs. The scores for each factor were standardized to have a mean of 0 and a standard deviation of 1, allowing for comparability between the factors.

The first factor (Factor 1) is characterized by higher loadings for pandemics (0.51), prolonged power outages (0.72), armed attacks (0.52), and extreme heat (0.55), suggesting it captures a general perception of large‐scale or systemic risks. Wildfires loaded equally on both factors (0.43 on Factor 1 and 0.43 on Factor 2), indicating it may reflect both systemic and environmental concerns. The second factor (Factor 2) is defined by a strong loading for floods (0.96), suggesting it represents more localized and environmental risks.

The two factors identified through the exploratory factor analysis both had an acceptable internal consistency. Cronbach's alpha was 0.737 for the first factor, reflecting systemic risk perceptions, and 0.695 for the second factor, reflecting local and environmental risk perceptions. The factor scores were subsequently included as independent variables in regression models.

### 4.4 Individual Level Control Variables

Our models also include several background variables that control for the individual level characteristics of the respondents. Education is accounted for by a categorical variable: “Primary school,” “High school,” “University.” Age is also accounted for by a categorical variable (“16–29,” “30–44,” “45–64,” “65–89”). Moreover, the model includes binary variables for the respondent's sex (“Male,” “Female”), if the respondent is a parent with children living at home (“Not a parent,” “Parent”), if the respondent is married or in a registered partnership (“Married/partnership,” “Not married/partnership”), and if the respondent has a foreign background, meaning that he/she was born abroad or that both parents were born abroad (“Foreign background,” “Not foreign background”). We also included a numerical variable for the respondents’ income. Table 1 shows the descriptive statistics of the various variables used in our models.

**TABLE 1:** Descriptive statistics for the various variables in the analysis.

	Type	Mean	SD	Median	Percent
Resiliance index	Scale	2.68	1.18	2.5	
Preparedness index	Scale	1.78	1.92	1	
DeSO [rural areas]	Categorical				17.9
DeSO [towns]	Categorical				7.3
DeSO [municipal centers]	Categorical				45.5
DeSO [metropolitan areas]	Categorical				29.3
RegSO [significant challenges]	Categorical				3.2
RegSO [moderate challenges]	Categorical				5.2
RegSO [mixed conditions]	Categorical				23.2
RegSO [favorable conditions]	Categorical				57.8
RegSO [very favorable conditions]	Categorical				10.6
Factor 1 (systematic risks)	Scale	0	0.83	0.02	
Factor 2 (localized risks)	Scale	0	0.98	−0.08	
Income	Scale	3977	4814	3334	
Age [18–29]	Categorical				3.6
Age [30–44]	Categorical				14.3
Age [45–64]	Categorical				34.0
Age [65–89]	Categorical				48.2
Gender [man]	Categorical				47.8
Gender [women]	Categorical				52.2
Foreign background [no]	Categorical				88.3
Foreign background [yes]	Categorical				11.7
Parent [not a parent]	Categorical				78.3
Parent [parent]	Categorical				21.7
Education [primary school]	Categorical				8.9
Education [high school]	Categorical				44.9
Education [university]	Categorical				46.2
Household [married/partnership]	Categorical				52.8
Household [not married/partnership]	Categorical				48.2

### 4.5 Analytical Strategy

To examine the relationship between individual preparedness and resilience and contextual as well as individual factors, we employ descriptive statistics and multilevel linear regression models. Because individuals are grouped within multiple geographic contexts—DeSO neighborhoods within RegSO areas, and RegSO areas within municipalities—observations are not fully independent. Multilevel modeling accounts for this nested data structure by estimating effects at each level, thereby avoiding biased standard errors and improving inference. To assess whether risk perception helps explain these associations (i.e., a pattern consistent with partial mediation), we estimate the regression models in two steps. In the first step, we include only individual sociodemographic variables and contextual variables to assess baseline associations. In the second step, we add individual‐level risk perception factors to evaluate whether any attenuation of contextual coefficients is consistent with partial mediation; because measures are contemporaneous and cross‐sectional, we do not make causal mediation claims. This stepwise approach helps clarify the incremental contribution of perceived risk to differences in preparedness behaviors and anticipated resilience.

## 5 Results

### 5.1 Resilience Index

**FIGURE 2:**
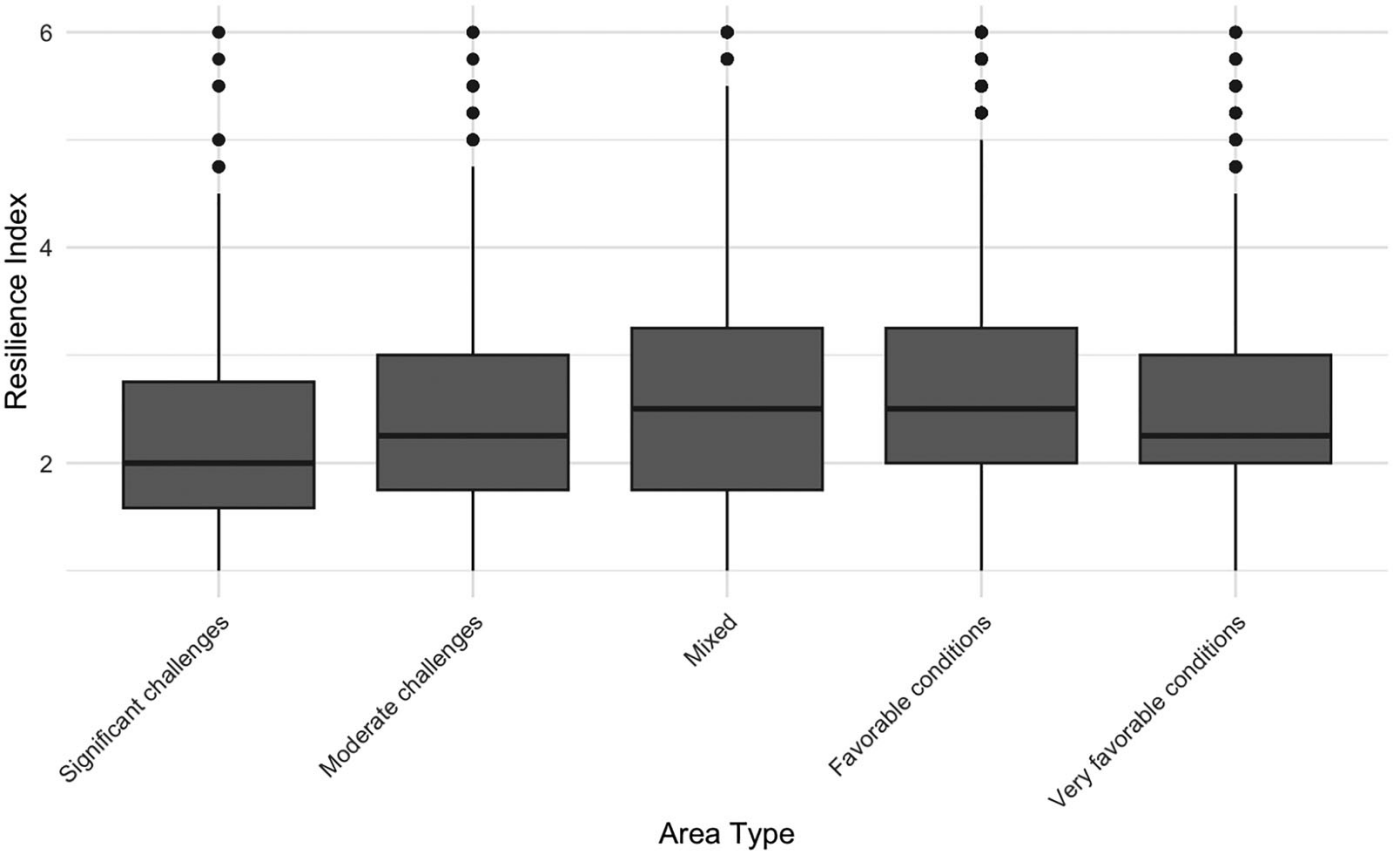
Perceived crisis resilience across areas with varying socioeconomic challenges.

To examine variations in resilience—defined as the estimated duration respondents believe they could manage without public services—we began with a descriptive quantitative analysis. Figure 2 illustrates differences across areas according to their score on the Segregation Barometer. Respondents from areas with mixed and favorable socioeconomic conditions reported slightly longer estimated durations compared to those from areas facing significant challenges, moderate challenges, or very favorable conditions.

**FIGURE 3:**
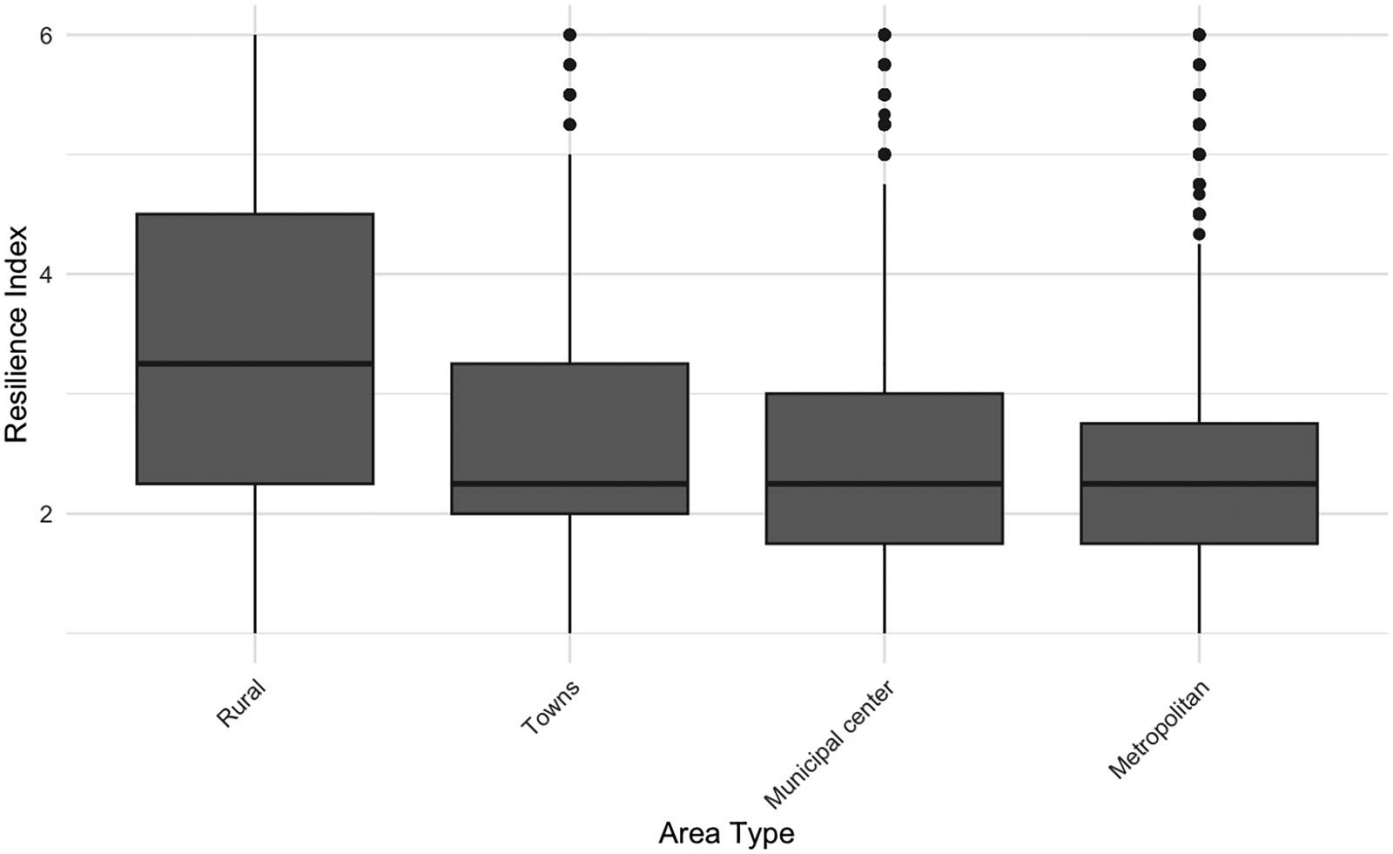
Perceived crisis resilience across urban and rural areas.

When comparing urban and rural areas, the disparities become even more pronounced (see Figure 3). Respondents from rural areas anticipate coping significantly longer without public services compared to those in other types of areas. On average, the resilience index scores for rural respondents fall between the responses “1–2 weeks” and “2–4 weeks.” Meanwhile, respondents in towns outside municipal centers estimate they would manage to fend for themselves slightly longer than those in municipal centers, who in turn expect to cope for a slightly longer period than respondents living in the three metropolitan areas. Consequently, metropolitan residents are expected to have the shortest resilience. It is important to note that residents of the three major metropolitan municipalities represent about a fourth of the entire Swedish population, excluding the neighboring suburban municipalities.

Multilevel linear regression models with random intercepts were used to test whether the context in which respondents live influences their estimated levels of resilience. To assess the potential mediating role of risk perception, the analysis was conducted in two steps. The first model includes only contextual and individual‐level background variables, whereas the second adds risk perception factors. Table 2 presents the results from both models.

**TABLE 2:** Multilevel linear regression results for the resilience index (stepwise models with and without risk perception factors.

	Model 1			Model 2		
Predictors	Estimates	CI	*p*	Estimates	CI	*p*
(Intercept)	3.32	3.14–3.50	**<0.001**	3.33	3.15–3.51	**<0.001**
Age [18–29 years] (ref.)						
Age [30–44 years]	−0.10	−0.21 to 0.02	0.108	−0.09	−0.21 to 0.02	0.123
Age [45–64 years]	−0.13	−0.24 to −0.02	**0.016**	−0.13	−0.24 to −0.02	**0.017**
Age [65–89 years]	−0.38	−0.49 to −0.27	**<0.001**	−0.38	−0.49 to −0.27	**<0.001**
Education [primary school] (ref.)						
Education [high school]	0.11	0.04–0.18	**0.002**	0.11	0.04–0.18	**0.002**
Education [University]	0.11	0.04–0.19	**0.002**	0.11	0.04–0.18	**0.002**
Income	0.00	−0.00 to 0.00	0.260	0.00	−0.00 to 0.00	0.275
Gender [woman] (ref.)						
Gender [man]	0.24	0.20–0.27	**<0.001**	0.22	0.18–0.26	**<0.001**
Household [not married] (ref.)						
Household [married]	0.01	−0.04 to 0.05	0.805	0.00	−0.04 to 0.04	0.830
Children [no] (ref.)						
Children [yes]	−0.04	−0.09 to 0.02	0.178	−0.04	−0.09 to 0.02	0.191
Foreign background [no] (ref.)						
Foreign background [yes]	−0.04	−0.10 to 0.02	0.162	−0.04	−0.10 to 0.02	0.158
Factor National risks				−0.01	−0.03 to 0.02	0.463
Factor local risks				−0.03	−0.05 to −0.01	**0.003**
Rurality [rural areas] (ref.)						
Rurality [towns]	−0.70	−0.79 to −0.61	**<0.001**	−0.70	−0.79 to −0.61	**<0.001**
Rurality [municipal centers]	−0.79	−0.85 to −0.73	**<0.001**	−0.79	−0.86 to −0.73	**<0.001**
Rurality [metropolitan]	−0.95	−1.17 to −0.74	**<0.001**	−0.96	−1.18 to −0.74	**<0.001**
SES context [significant challenges] (ref.)						
SES context [moderate challenges]	0.07	−0.09 to 0.22	0.396	0.07	−0.08 to 0.22	0.393
SES context [mixed conditions]	0.11	−0.01 to 0.24	0.076	0.11	−0.01 to 0.24	0.079
SES context [favorable conditions]	0.09	−0.03 to 0.21	0.151	0.09	−0.03 to 0.21	0.153
SES context [very favorable conditions]	0.13	−0.01 to 0.27	0.066	0.13	−0.01 to 0.27	0.072
**Random effects**						
*σ* ^2^	1.18			1.18		
*τ*_00 RegSOkod_2023_	0.02			0.02		
*τ*_00 Kommun_	0.04			0.04		
ICC	0.04			0.04		
*N*_Kommun_	281			281		
*N*_RegSOkod_2023_	2316			2316		
Observations	12,574			12,574		
Marginal *R* ^2^/Conditional *R* ^2^	0.100/0.140			0.101/0.141		

*Note*: Bold values indicate statistical significance at the five percent level (*p* < 0.05).

Confirming the findings from the descriptive analysis, the results from the first regression model indicate that people living in more urbanized areas have lower resilience scores compared to those in rural areas. Specifically, individuals in towns have significantly lower scores than those in rural areas, with an estimated difference of −0.70 (*p* < 0.001). Those living in the center of municipalities have even lower scores (*b* = −0.79, *p* < 0.001), whereas respondents in metropolitan areas exhibit the lowest scores (*b* = −0.95, *p* < 0.001). However, the socioeconomic conditions of the neighborhoods does not show any significant associations with individual resilience levels.

Regarding individual‐level predictors, age shows a clear pattern, with older respondents having a lower resilience index score all else being equal. Education is also positively associated with resilience; respondents with either a secondary degree or university education score slightly higher than those without secondary education (both *b* = 0.11 and *p* = 0.002). Men have a higher resilience index than women (*b* = 0.24, *p* < 0.001) all else being equal, whereas marital status (*p* = 0.805), the presence of children at home (*p* = 0.178), and foreign background (*p* = 0.162) do not significantly affect the respondents’ perceived resilience.

After adding the risk factors in the second model, the coefficients remained largely unchanged. The factor capturing localized risks shows a small but statistically significant negative association with perceived resilience (*b* = −0.03, *p* = 0.003), indicating that individuals who perceive a higher likelihood of being affected by local risks also expect to cope for a slightly shorter time during a crisis. However, the limited change in coefficients between the two models is consistent with little or no mediation by risk perception in the association between place of residence and resilience.

### 5.2 Preparedness Index

**FIGURE 4:**
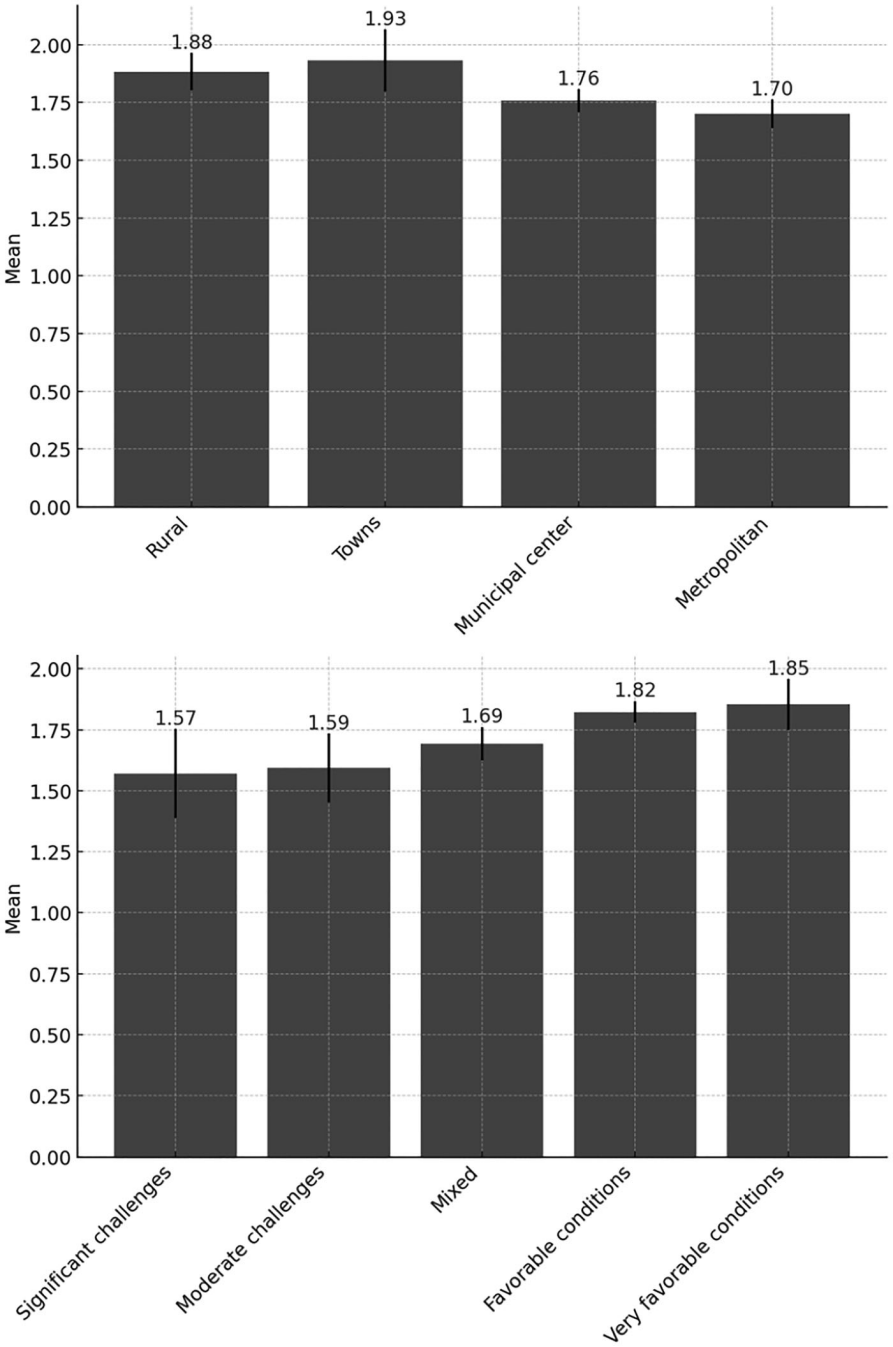
Preparedness index across urban–rural and socioeconomic area types.

The preparedness index measures the number of actions an individual has taken to prepare for a crisis or disaster. Similar to the resilience index, we began with a descriptive quantitative analysis to explore differences across different areas. Figure 4 highlights variations in preparedness across areas with differing socioeconomic conditions and levels of urbanity/rurality.

The results indicate that residents in wealthier areas generally have taken more actions to prepare for a crisis or disaster, although the differences are relatively modest. In areas facing significant challenges, respondents have taken an average of 1.57 actions, compared to 1.85 actions in areas with very favorable conditions. Concerning differences between rural and urban areas, respondents in rural areas and towns have taken slightly more actions on average, with scores of 1.88 and 1.93, respectively, compared to those in municipal centers and metropolitan areas, who scored 1.76 and 1.70, respectively.

To examine whether the context in which respondents live influences individual preparedness, we also estimated multilevel linear regression models in two steps. The first model includes contextual and individual‐level background variables, whereas the second adds the risk perception factors (see Table 3). In the Supporting Information section, we also present results from a zero‐inflated negative binomial multilevel regression model using the same variables (see Table A1). We opted to include the zero‐inflated negative binomial model in the Supporting Information section and use the multilevel linear regression in the main article because the latter offers results that are easier to interpret. Meanwhile, the former serves as a robustness check to validate our findings, considering the count nature of the data. Importantly, the results from both models are consistent, further substantiating the robustness of our conclusions.

**TABLE 3:** Multilevel linear regression results for the preparedness index (stepwise models with and without risk perception factors.

	Model 1			Model 2		
Predictors	Estimates	CI	*p*	Estimates	CI	*p*
(Intercept)	1.66	1.36–1.96	**<0.001**	1.47	1.18–1.77	**<0.001**
Age [18–29 years] (ref.)						
Age [30–44 years]	0.19	−0.01 to 0.39	0.063	0.12	−0.08 to 0.32	0.226
Age [45–64 years]	−0.01	−0.20 to 0.18	0.901	−0.04	−0.23 to 0.15	0.674
Age [65–89 years]	−0.19	−0.38 to −0.00	**0.044**	−0.18	−0.37 to 0.00	0.055
Education [primary school] (ref.)						
Education [high school]	0.12	0.00–0.25	**0.049**	0.13	0.01–0.25	**0.035**
Education [University]	0.28	0.15–0.40	**<0.001**	0.27	0.14–0.39	**<0.001**
Income	0.00	−0.00 to 0.00	0.225	0.00	−0.00 to 0.00	0.162
Gender [woman] (ref.)						
Gender [man]	−0.04	−0.11 to 0.03	0.265	0.15	0.09–0.22	**<0.001**
Household [not married] (ref.)						
Household [married]	0.14	0.07–0.21	**<0.001**	0.14	0.07–0.21	**<0.001**
Children [no] (ref.)						
Children [yes]	0.09	−0.01 to 0.18	0.074	0.09	−0.00 to 0.18	0.064
Foreign background [no] (ref.)						
Foreign background [yes]	−0.21	−0.31 to −0.10	**<0.001**	−0.18	−0.29 to −0.08	**<0.001**
Factor national risks				0.43	0.39–0.47	**<0.001**
Factor local risks				0.04	0.00–0.07	**0.027**
Rurality [rural areas] (ref.)						
Rurality [towns]	0.01	−0.15 to 0.16	0.918	0.07	−0.08 to 0.22	0.376
Rurality [municipal centers]	−0.14	−0.24 to −0.04	**0.008**	−0.03	−0.13 to 0.07	0.598
Rurality [metropolitan]	−0.21	−0.39 to −0.03	**0.020**	−0.06	−0.23 to 0.11	0.493
SES context [significant challenges] (ref.)						
SES context [moderate challenges]	−0.06	−0.31 to 0.19	0.619	−0.08	−0.33 to 0.17	0.518
SES context [mixed conditions]	0.02	−0.19 to 0.23	0.878	0.01	−0.20 to 0.22	0.910
SES context [favorable conditions]	0.10	−0.10 to 0.31	0.332	0.10	−0.10 to 0.30	0.343
SES context [very favorable conditions]	0.08	−0.15 to 0.31	0.479	0.10	−0.13 to 0.32	0.391
**Random effects**						
*σ* ^2^	3.59			3.47		
*τ*_00 RegSOkod_2023_	0.03			0.03		
*τ*_00 Municipality_	0.01			0.01		
ICC	0.01			0.01		
*N*_Kommun_	281			281		
*N*_RegSOkod_2023_	2316			2316		
Observations	12,574			12,574		
Marginal *R* ^2^/Conditional *R* ^2^	0.016/0.027			0.048/0.059		

*Note*: Bold values indicate statistical significance at the five percent level (*p* < 0.05).

As shown in Table 3, results from the first model—without the risk perception variables—indicate that the context in which individuals reside does influence the number of actions taken to prepare for disasters and crises. Specifically, respondents living in municipal centers (*b* = −0.14, *p* = 0.008) and metropolitan areas (*b* = −0.21, *p* = 0.020) report fewer preparedness actions compared to those in rural areas.

At this stage, gender is not significantly associated with preparedness (*b* = −0.04, *p* = 0.265), whereas education, marital status, and foreign background show statistically significant effects. Individuals with higher levels of education, particularly those with a university degree (*b* = 0.28, *p* < 0.001), report taking more preparedness actions than those with only primary schooling. Being married or in a registered partnership is positively associated with preparedness (*b* = 0.14, *p* < 0.001). Respondents with a foreign background, on the other hand, report significantly fewer preparedness actions than those without (*b* = −0.21, *p* < 0.001).

In the second model, which includes the two risk perception factors, the coefficients for municipal centers and metropolitan areas attenuate and become statistically nonsignificant, a pattern consistent with partial mediation by risk perception. Similarly, the previously nonsignificant gender effect becomes significant when risk perception is accounted for: Men are more likely than women to have prepared (*b* = 0.15, *p* < 0.001). This shift indicates a suppression effect, where differences in risk perception between men and women were initially masking underlying behavioral differences. Women generally perceive higher levels of risk, which tends to increase preparedness. Once risk perception is held constant, it becomes clear that men report taking more preparedness actions than women with similar levels of perceived risk. Individuals who perceive a higher risk of being affected by various national (systematic) risks have taken more actions to prepare (*b* = 0.43, *p* < 0.001), whereas localized risks have a smaller but still significant association (*b* = 0.04, *p* = 0.027). The effects of education, marital status, and foreign background remain stable between models, suggesting that their influence on preparedness is not mediated by perceived risk.

## 6 Discussion

This study set out to investigate two potential context effects on individual levels of crisis preparedness and resilience: socioeconomic differences across contexts and urban versus rural contexts. By employing multilevel analysis, this study aimed to assess the impact of these specific living contexts on individual resilience and preparedness. Our findings reveal significant context effects attributable to rural environments on individuals’ resilience and preparedness levels. These results are consistent with prior studies that have examined the relationship between preparedness and rurality in Sweden (e.g., Guldåker 2009). Individuals living in municipal centers and metropolitan areas expect to be less resilient in the face of a crisis compared to rural residents, and they have also taken fewer actions to prepare.

When we included the risk perception variable in the preparedness model, the differences across urban and rural contexts were no longer statistically significant. This pattern is consistent with risk perception functioning as a mediating variable, indicating that a heightened sense of vulnerability and perceived risk may help explain the higher levels of preparedness observed among rural residents. These results also corroborate other studies on preparedness for more specific risks, such as residential fires, that suggest that rural residents living in areas with longer rescue service response times (and thus higher risks) do indeed have more fire protection measures than their urban counterparts (Henrekson et al. 2025). This pattern supports the idea that individuals act rationally, adapting their preparedness behaviors in response to perceived levels of risk.

We hypothesize that the greater sense of resilience among rural residents stems from the denser social networks built on stronger ties, deeper local knowledge, and better access to community support—factors that are conducive to a greater sense of security (Cutter et al. 2016). Additionally, individuals in rural areas may naturally have more food supplies at home, due to larger living spaces and greater distances to stores, and have alternative heating sources and their own water wells (Guldåker 2016; Kohler et al. 2020). This sense of resilience may, however, be misplaced if the anticipated support from social networks fails and is not complemented by residents taking proactive steps to prepare themselves. Moreover, the age structure of many rural communities, characterized by a higher proportion of older adult residents, may contribute to a greater aggregate vulnerability of rural communities as many depend on home care services.

Our analyses did not uncover any statistically significant context effects from residing in socioeconomically disadvantaged districts. However, this should not be interpreted as an indication that socioeconomic resources are unimportant (cf., Bergstrand et al. 2015; Norris et al. 2008). On the contrary, socioeconomic factors play a critical role in explaining individual variations in crisis preparedness. However, our analyses indicate that the influence of living in either disadvantaged or advantaged communities does not have any additional statistical impact on individual preparedness levels. That is, although socioeconomic resources are crucial, we could not identify any additional contextual effects. Hence, we interpret the varying levels of preparedness across neighborhoods with different types of socioeconomic resources as a result of compositional effects (i.e., that individuals with similar status live next to each other). Nevertheless, and in line with previous studies (e.g., Bergstrand et al. 2015; Cong et al. 2023; de Oliveira Mendes 2009; Norris et al. 2008; Ran et al. 2020; Yong et al. 2020), we conclude that public authorities must consider the spatial clustering of groups when planning for crisis preparedness and community resilience, as it may amplify vulnerabilities at the community level. This implies that preparedness campaigns, resource allocation, and local risk communication strategies should be designed with spatial differences in mind—targeting not only individuals but also entire communities.

Our study has limitations, the most significant being its reliance on survey data, which means we do not observe actions directly but rather rely on self‐reported actions and subjective estimates of household resilience during crises. There is an ongoing methodological debate about people's tendency to overestimate their own abilities in various statistical surveys. At the same time, research suggests that people are often better prepared for crises than they perceive themselves to be (Heidenstrøm and Kvarnlöf 2018; Wall and Kvarnlöf 2019; Kvarnlöf and Wall 2021). These complexities highlight the importance of combining self‐reported data with other methodologies in future research to gain a more comprehensive understanding of preparedness behaviors and resilience.

## 7 Conclusion

In the present study, we examined how levels of preparedness differed across socioeconomic contexts and urban versus rural living environments in Sweden. Our results show that rural residents both perceive themselves as more resilient and have taken more actions to prepare for disasters and crises. However, when differences in how people perceive risks are taken into account, the gap in preparedness between urban and rural residents largely attenuates and is no longer statistically significant. This pattern is consistent with risk perception playing a mediating role in shaping preparedness for crises or disasters. Additionally, although we observed descriptive differences in preparedness between contexts with varying socioeconomic conditions, these differences disappeared when controlling for individual‐level variables, suggesting they are driven by compositional effects rather than context effects.

Overall, this study broadens the understanding of how local contexts influence preparedness and highlights the complexity of these relationships. The findings underscore the need for tailored local strategies that consider both perceived resilience and actual preparedness levels within diverse community settings.

## Conflicts of Interest

The authors declare no conflicts of interest.

## Supporting information




**Supporting Figure A1**: Illustration of the survey data structure
**Supporting Table A1**: Zero‐inflated negative binomial regression results for the preparedness index

## Data Availability

The data that support the findings of this study are available on request from the corresponding author.
