# Supplementary material for: Resilience and Preparedness Across Place: A Multilevel Analysis of Urban–Rural and Socioeconomic Divides
Source: Risk Anal. 2025 Nov 21;45(12):4933–46. doi: 10.1111/risa.70155 (PMC12747688; doi:10.1111/risa.70155)
Supplement: Supplementary file 1 — Supporting Figure A1: Illustration of the survey data structure Supporting Table A1: Zero‐inflated negative binomial regression results for the preparedness index [file RISA-45-4933-s001.docx]

**Supplementary material**

Figure A1 Illustration of the survey data structure


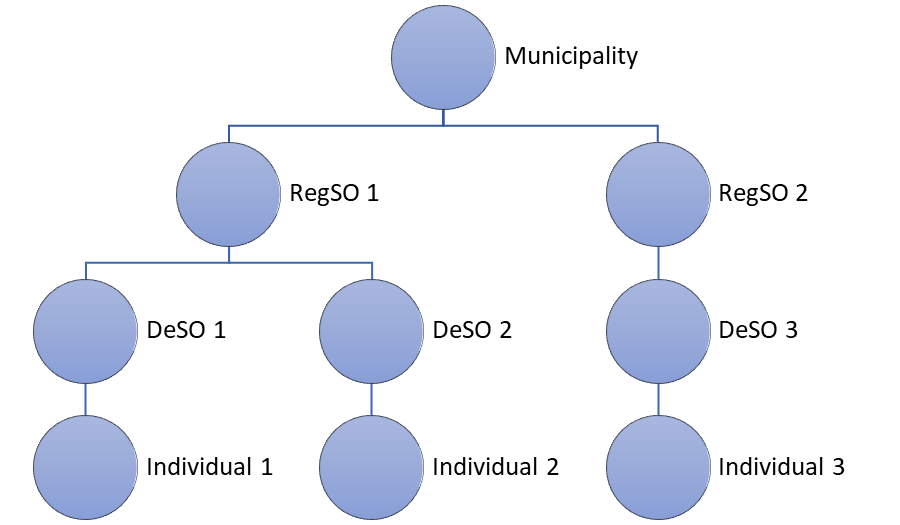


Table A1. Zero-inflated negative binomial regression results for the preparedness index

| *Predictors* | *Incidence Rate Ratios* | *CI* | *p* |
| --- | --- | --- | --- |
| (Intercept) | 2.01 | 1.72 – 2.35 | **<0.001** |
| Age [18–29 years] (ref.) |  |  |  |
| Age [30–44 years] | 1.06 | 0.96 – 1.17 | 0.281 |
| Age [45–64 years] | 1.01 | 0.92 – 1.12 | 0.774 |
| Age [65–89 years] | 0.90 | 0.82 – 1.00 | **0.041** |
| Education [Primary school] (ref.) | |  |  |
| Education [High school] | 1.05 | 0.98 – 1.12 | 0.146 |
| Education [University] | 1.12 | 1.05 – 1.20 | **0.001** |
| Income | 1.00 | 1.00 – 1.00 | **0.040** |
| Gender [Woman] (ref.) |  |  |  |
| Gender [Man] | 1.09 | 1.05 – 1.13 | **<0.001** |
| Household [Not married] (ref.) |  |  |  |
| Household [Married] | 1.07 | 1.03 – 1.10 | **<0.001** |
| Children [no] (ref.) |  |  |  |
| Children [yes] | 1.05 | 1.00 – 1.10 | **0.040** |
| Foreign background [no] (ref.) |  |  |  |
| Foreign background [yes] | 0.92 | 0.87 – 0.97 | **0.004** |
| Factor National risks | 1.19 | 1.17 – 1.22 | **<0.001** |
| Factor Local risks | 1.01 | 1.00 – 1.03 | 0.136 |
| Rurality [Rural areas] (ref.) |  |  |  |
| Rurality [Towns] | 1.05 | 0.98 – 1.14 | 0.175 |
| Rurality [Municipal centers] | 0.99 | 0.95 – 1.04 | 0.791 |
| Rurality [Metropolitan] | 0.99 | 0.92 – 1.06 | 0.704 |
| SES context [Significant challenges] (ref.) | |  |  |
| SES context [Moderate challenges] | 0.94 | 0.83 – 1.08 | 0.386 |
| SES context [Mixed conditions] | 1.02 | 0.91 – 1.14 | 0.710 |
| SES context [Favorable conditions] | 1.05 | 0.95 – 1.17 | 0.350 |
| SES context [Very favorable conditions] | 1.04 | 0.92 – 1.17 | 0.547 |
| **Zero-Inflated Model** |  |  |  |
| (Intercept) | 0.37 | 0.35 – 0.39 | **<0.001** |
| **Random Effects** |  |  |  |
| σ^2^ | 0.49 |  |  |
| τ_00_ _Kommun_ | 0.00 |  |  |
| τ_00_ _RegSOkod_2023_ | 0.01 |  |  |
| ICC | 0.02 |  |  |
| N _Kommun_ | 281 |  |  |
| N _RegSOkod_2023_ | 2316 |  |  |
| Observations | 12574 |  |  |
| Marginal R^2^ / Conditional R^2^ | 0.065 / 0.080 | |  |
